# A Wavelet-Based Steganographic Method for Text Hiding in an Audio Signal

**DOI:** 10.3390/s22155832

**Published:** 2022-08-04

**Authors:** Olga Veselska, Oleksandr Lavrynenko, Roman Odarchenko, Maksym Zaliskyi, Denys Bakhtiiarov, Mikolaj Karpinski, Stanislaw Rajba

**Affiliations:** 1 Department of Computer Science and Automatics, University of Bielsko-Biala, 43-309 Bielsko-Biala, Poland; 2 Department of Telecommunication and Radio-Electronic Systems, National Aviation University, 03058 Kyiv, Ukraine

**Keywords:** audio signal, text information masking, steganographic encoder, spectrum analysis, wavelet transform, wavelet coefficients, orthogonal wavelet filters

## Abstract

The developed method of steganographic hiding of text information in an audio signal based on the wavelet transform acquires a deep meaning in the conditions of the use by an attacker of deliberate unauthorized manipulations with a steganocoded audio signal to distort the text information embedded in it. Thus, increasing the robustness of the stego-system by compressing the steganocoded audio signal subject to the preservation of the integrity of text information, taking into account the features of the psychophysiological model of sound perception, is the main objective of this scientific research. The task of this scientific research is effectively solved using a multilevel discrete wavelet transform using adaptive block normalization of text information with subsequent recursive embedding in the low-frequency component of the audio signal and further scalar product of the obtained coefficients with the Daubechies wavelet filters. The results of the obtained experimental studies confirm the hypothesis, namely that it is proposed to use recursive embedding in the low-frequency component (approximating wavelet coefficients) followed by their scalar product with wavelet filters at each level of the wavelet decomposition, which will increase the average power of hidden data. It should be noted that upon analyzing the existing method, which is based on embedding text information in the high-frequency component (detailed wavelet coefficients), at the last level of the wavelet decomposition, we obtained the limit CR = 6, and in the developed, CR = 20, with full integrity of the text information in both cases. Therefore, the resistance of the stego-system is increased by 3.3 times to deliberate or passive compression of the audio signal in order to distort the embedded text information.

## 1. Introduction

Recently, scientific research in the field of wireless acoustic sensor networks solves very important technical problems. Many areas have been covered, such as self-localization of acoustic sensors, recognition and coding of audio signals, active noise control, and localization of sound sources [1,2].

This paper considers another important area in acoustic sensory systems, information security, which will allow use of a highly redundant audio signal that is received from acoustic sensors as a container for hiding text information in it, so that the classical problem of audio steganography is solved. It will also be quite relevant to apply the developed method in voice messengers, where a fake voice message is transmitted that hides a true text message. In this case, the attacker will not be able to recognize the essence of the hidden correspondence of users, and if we assume that the microphone of a mobile device will act as an acoustic sensor, then it is possible to mask hidden correspondence against the background of another audio conference in real time, which also can confuse the attacker. It is necessary to remember the features of text recognition systems against the background of multimedia information (images, video), which is obtained from video sensors, where the recognized text can also be hidden in the audio signal of acoustic sensor networks.

It should be noted that if we slightly modify the developed method at the stage of processing hidden information before integrating it into the audio signal (adapt the method to another type of information), then it can be easily used not only for hiding text information, but also for hiding signal parameters (object recognition features), which are the result of the analysis, processing, and classification of information received from different types of network sensors (video and audio sensors). This type of hidden information is very common today in computer vision, speech, and video recognition. In this case, it is not the carrier signal itself (audio, video, images) that is subject to hiding in the audio signal, but its recognition features, depending on the specific classification task being solved. For example, the semantic parameters of speech or the biometric features of the voice can be hidden in the audio signal; if we are talking about recognizing video information or images, then there is an opportunity to hide the parameters that characterize the tracking of moving objects in time, identification of a person by photo, optical character recognition, and other such signal parameters.

To ensure the effective hiding of text information in an audio signal, a deep understanding of their amplitude–frequency characteristics [3] is required. This is because many factors will depend on the correct analysis of where in the amplitude–frequency component text information is to be integrated. The main ones are the effectiveness of the hiding (masking) itself, as well as the resistance of the steganocodec to audio container transcoding. A fundamental understanding of the spectral features of audio signals [4] will allow balancing between increasing the efficiency of hiding text information in an audio container and resistance to various compression algorithms of a steganographic audio file.

Therefore, the question arises whether the secret text information will be preserved without distortion when re-transcoding the steganographic audio file, and if so, what is the maximum value of the compression ratio at which the secret information maintains integrity? In particular, this question prompted the authors to write this article and develop one of the methods for steganographic hiding of text information in an audio signal [5,6,7], which will allow for answering the contradictions that have arisen using modern methods of digital audio signal processing and spectral analysis methods.

### 1.1. Problem Statement

The developed method of steganographic hiding of text information in an audio signal based on the wavelet transform [8] acquires a deep meaning in the conditions of the use by an attacker of deliberate unauthorized manipulations with a steganocoded audio signal to distort the text information embedded in it; that is, to make its semantic constructions illegible. The main form of these manipulations is the use of various algorithms for compressing the audio signal [9,10], but not to remove its uninformative components, which, according to the human psychophysiological model of sound perception, are beyond the threshold of audibility, and to remove the text information hidden in the audio signal by deliberately introducing distortions by the compression algorithm.

Thus, increasing the robustness of the stego-system to compression (reducing redundancy) of the steganocoded audio signal [11,12] subject to the preservation of the integrity of text information (genuine semantic structures), taking into account the features of the psychophysiological model of sound perception (hiding the very fact of text transmission by masking in acoustic signals), is the main objective of this scientific research.

### 1.2. Analysis of Existing Research and Formation of a Scientific Hypothesis

The task of this scientific research is effectively solved using a multilevel discrete wavelet transform [8,13] based on adaptive block normalization of text information with subsequent recursive embedding in the low-frequency component of the audio signal and further scalar product of the obtained coefficients with the Daubechies wavelet filters [14,15], which is a new approach in the field of steganography that makes the stego-system more resistant to transcoding. The difference between the developed method and the existing ones is that in existing steganographic methods of information hiding based on wavelet transform [16,17,18,19,20], text information is usually embedded in the high-frequency wavelet coefficients (HFWC) at the last level of the wavelet decomposition, and in the developed method, it is proposed to use recursive embedding in the low-frequency wavelet coefficients (LFWC) followed by scalar product with orthogonal Daubechies filters at each level of the wavelet decomposition, which allows for increasing the average power of hidden data. This will increase the critical compression threshold of the steganocoded audio signal, at which the text will begin to distort (the transmitted message will be different from the received).

The formalization of the mentioned statements is as follows:

(1) An existing method that is used in many studies [16,17,18,19,20] in different configurations, where for the most part, we may apply the idea of text integration according to the expression $T_{1\ldots L}\to Y_{j}$ in Formula (1):

(1) $${A^{\prime }}_{k}=\sum_{j=\max\left(1,\;k+1-l_{F}\right)}^{\min\left(k,\;2l_{Z}-1\right)}\left(Z_{j}\right)↑2\cdot R_{i}+\sum_{j=\max\left(1,\;k+1-l_{F}\right)}^{\min\left(k,\;2l_{Z}-1\right)}\left(T_{1\ldots L}\to Y_{j}\right)↑2\cdot W_{i},\;k=1,\;\ldots ,\;2l_{Z}-1+l_{F}-1,$$

(2) $$Z_{k}=\left(\sum_{j=\max\left(1,\;k+1-l_{F}\right)}^{\min\left(k,\;l_{A}\right)}A_{j}D_{i}\right)↓2,\;k=1,\;\ldots ,\;l_{A}+l_{F}-1,$$

(3) $$Y_{k}=\left(\sum_{j=\max\left(1,\;k+1-l_{F}\right)}^{\min\left(k,\;l_{A}\right)}A_{j}V_{i}\right)↓2,\;i=k+1-j,$$

(2) The proposed method differs significantly in the expression $\left(T_{1\ldots L-1}\to Z_{j}\right)D_{i}$ in Formula (5), which allows for increasing the average power of hidden text information due to the scalar product with the coefficients of the low-frequency wavelet filter $D_{i}$:

(4) $${A^{\prime }}_{k}=\sum_{j=\max\left(1,\;k+1-l_{F}\right)}^{\min\left(k,\;2l_{Z}-1\right)}\left(Z_{j}\right)↑2\cdot R_{i}+\sum_{j=\max\left(1,\;k+1-l_{F}\right)}^{\min\left(k,\;2l_{Z}-1\right)}\left(Y_{j}\right)↑2\cdot W_{i},\;k=1,\;\ldots ,\;2l_{Z}-1+l_{F}-1,$$

(5) $$Z_{k}=\left(\sum_{j=\max\left(1,\;k+1-l_{F}\right)}^{\min\left(k,\;l_{Z}\right)}\left(T_{1\ldots L-1}\to Z_{j}\right)D_{i}\right)↓2,\;k=1,\;\ldots ,\;l_{Z}+l_{F}-1,$$

(6) $$Y_{k}=\left(\sum_{j=\max\left(1,\;k+1-l_{F}\right)}^{\min\left(k,\;l_{Z}\right)}Z_{j}V_{i}\right)↓2,\;i=k+1-j,$$

where A, $A^{\prime }$ are input and output audio signals with number of samples $l_{A}$; T, $T^{\prime }$ are input and output texts divided into 1,2,…,L blocks depending on the number of wavelet decomposition levels L; $Z_{k}$, $Y_{k}$ are wavelet coefficients of low and high frequencies in quantity $l_{Z}$; D,V,R,W are Daubechies filters of the N-th order low and high frequencies for decomposition and reconstruction; ↓2, ↑2 are operations of double thinning and excess; → is a symbol used to logically explain the operation of integrating text information into wavelet coefficients.

The expression $\left(T_{1\ldots L-1}\to Z_{j}\right)D_{i}$ in Formula (5) shows that the integration → of blocks of text information $T_{1\ldots L-1}$ into wavelet coefficients $Z_{j}$ occurs at the levels of the wavelet decomposition 1,…,L−1 to their scalar product with a low-pass Daubechies filter $D_{i}$, as opposed to the expression $T_{1\ldots L}\to Y_{j}$ in Formula (1), where integration → into wavelet coefficients $Y_{j}$ occurs after the scalar product with the high-pass Daubechies filter $V_{i}$ (3).

Extraction of text information $T^{\prime }$ from an audio signal $A^{\prime }$ occurs recursively depending on the number of levels of the wavelet decomposition L according to Formulas (2), (3), (5), and (6) in the existing [16,17,18,19,20] and proposed approaches, respectively.

Thus, using the developed method, it is possible to allow an attacker to re-encode the audio signal with various lossy compression algorithms, but at the same time, the text information embedded in the audio signal maintains integrity. This statement is based on the fact that the current variety of existing compression algorithms [9,10,11,12] operates according to the same principle, namely, the elimination of the uninformative redundant component of the audio signal. Since the proposed method hides text information at medium frequencies and amplitudes of wavelet coefficients, and because this is its main feature, it can significantly increase the resistance of the stego-system to audio signal compression, taking into account the features of the psychophysiological model of sound perception. The only exceptions are those cases of completely deleting an audio file or applying critical compression with a complete loss of meaningful audio information. A quantitative assessment of the boundary values of critical compression occurrence will be obtained in an experimental study. Critical compression should be understood as the degree of compression at which text information is distorted or completely deleted (violation of semantic links) from the audio signal with a significant reduction in redundancy (compression). Then the main evaluation for the effectiveness of the proposed stego-system is the maximum degree of audio signal compression and the integrity of text information; that is, the highest compression ratio that maintains the full integrity of the semantic structures of the text.

Analysis of the literature [16,17,18,19,20,21,22,23,24,25,26] shows an almost complete absence of methods for embedding compression-resistant audio signals. One of the transformations that allows for such an embedding is the multilevel discrete wavelet transform, which has clear advantages in representing the local characteristics of the signal and takes into account the features of the psychophysiological model of sound perception. The proposed method increases the robustness of the stego-system to deliberate compression (elimination of highly informative features). We will show that the application of this approach in the development of the steganography algorithm, which is designed to achieve maximum robustness, can solve the main tasks of steganography, namely, minimization of introduced distortions and resistance to attacks by a passive intruder.

The next section is devoted to the presentation of all the main theoretical aspects of the proposed method, namely, (1) integrating text information into low-frequency wavelet coefficients of an audio signal followed by their scalar product with low-frequency and high-frequency orthogonal Daubechies wavelet filters for decomposition; (2) reconstructing of the audio signal with the text integrated into it by low-frequency and high-frequency wavelet coefficients; (3) extracting text information from low-frequency wavelet coefficients of the audio signal.

## 2. Presentation of the Proposed Method

**Figure 1:**
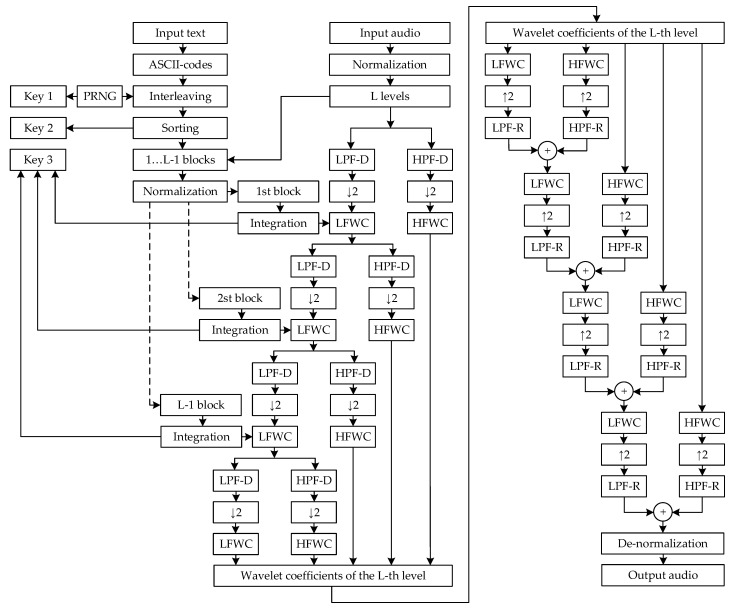
Structural diagram of the proposed method for integrating text information into an audio signal.

**Figure 2:**
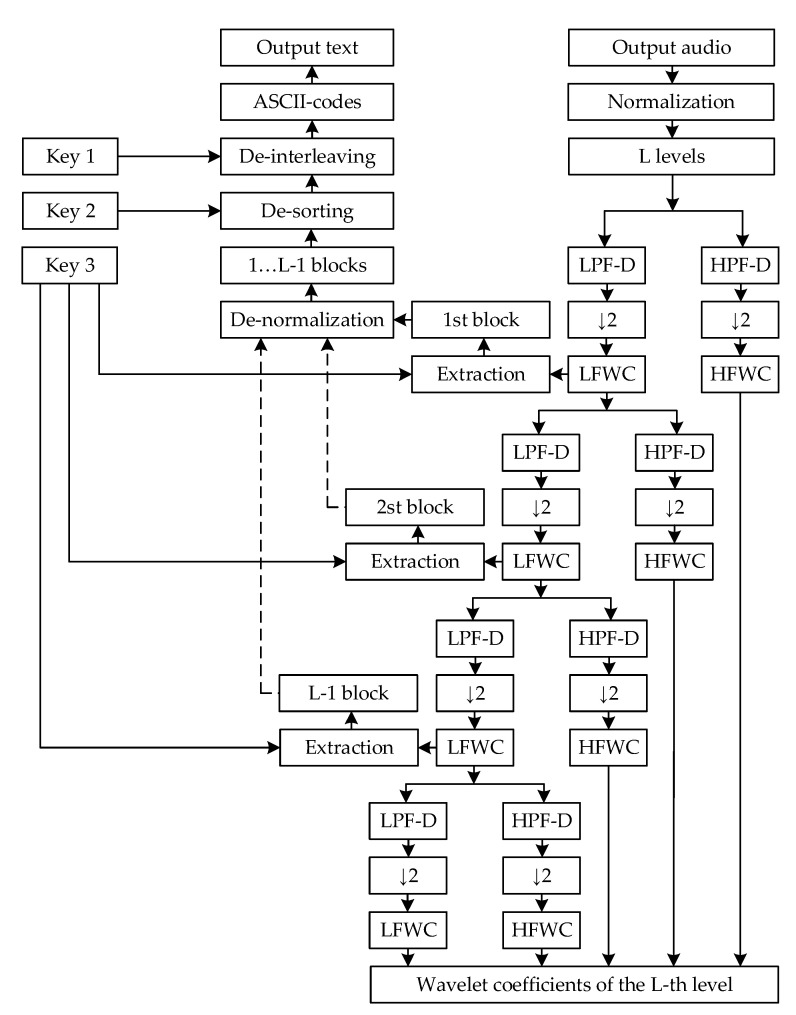
Structural diagram of the proposed method for extracting text information from an audio signal.

Structural diagrams of the developed method of steganographic protection of text information based on the wavelet transform are shown in Figure 1 and Figure 2. A detailed explanation of all the blocks on the diagram and their formal presentation are given below.

Any text information in English can be represented as an ASCII encoding, where all characters of the computer alphabet are numbered from 0 to 127, describing the ordinal number of a character in the binary number system of a seven-digit code from 0000000 to 1111111. Thus, we will form a set of numbers $S=\left\{0,1,2,\ldots ,127\right\}$ that correspond to each specific character according to the ASCII encoding. Then text information can be represented as a set $T=\left\{S_{i},S_{i},\ldots ,S_{i}\right\}$, which corresponds to a sequence of numbers $S_{i}$ from the set S, where the occurrence of each $S_{i}$ in the set T is determined by the sequence of characters in the text i.

So, given some text information $T_{1,\ldots ,l}$, where l is the total number of characters to be hidden in the audio signal, it is necessary to perform an interleaving operation to remove statistical dependencies between characters in the text. This operation is implemented using a pseudo-random number generator (PRNG), which forms a sequence of l uniformly distributed numbers in the range $\left(0;1\right)$.

Given a random variable, we often compute the expectation and variance, two important summary statistics. The expectation describes the average value, and the variance describes the spread (amount of variability) around the expectation.

Then, the mathematical expectation $m_{r}$ and variance $D_{r}$ of such a sequence, which consists of l pseudo-random numbers $r_{i}$, should tend → to the following values

(7) $$m_{r}=\frac{\sum_{i=1}^{l}r_{i}}{l}\to 0.5,$$

(8) $$D_{r}=\frac{\sum_{i=1}^{l}{\left(r_{i}-m_{r}\right)}^{2}}{l}\to \frac{1}{12}.$$

In order to shuffle the characters of the set $T_{1,\ldots ,l}$ in a pseudo-random way, it is necessary that the pseudo-random numbers $x_{1,\ldots ,l}$ that are generated by the PRNG are in the range $\left(1;l\right)$, which is different from $\left(0;1\right)$. Numbers in the range $\left(1;l\right)$ are equivalent to the indexes of each character of text information $T_{1,\ldots ,l}$.

To solve this problem, we can use the formula

(9) $$x_{1,\ldots ,l}=1+\left(l-1\right)\cdot r_{1,\ldots ,l},$$

where $r_{1,\ldots ,l}$—pseudo-random numbers from the range $\left(0;1\right)$.

**Figure 3:**
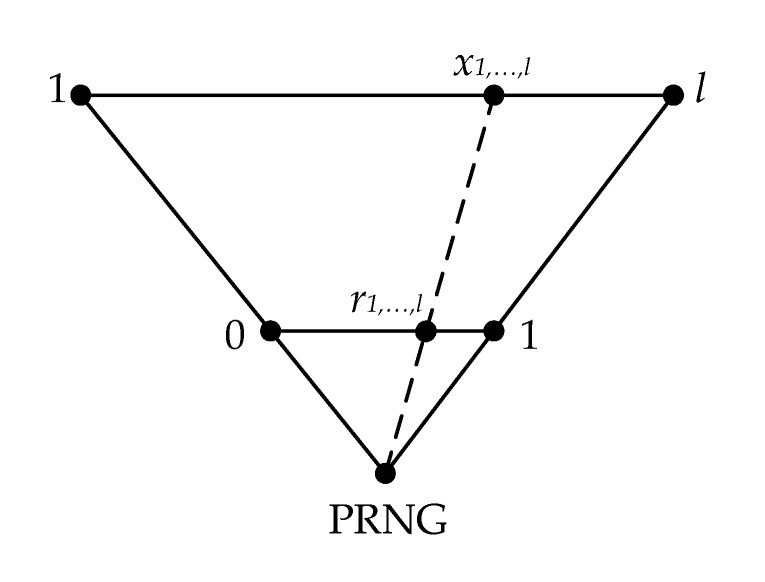
Scheme for converting pseudo-random numbers $r_{1,\ldots ,l}$ from the range $\left(0;1\right)$ into numbers $x_{1,\ldots ,l}$ with the range $\left(1;l\right)$.

The correctness of this transform is described as follows

(10) $$\frac{r_{1,\ldots ,l}-0}{1-0}=\frac{x_{1,\ldots ,l}-1}{l-1}\Leftrightarrow r_{1,\ldots ,l}=\frac{x_{1,\ldots ,l}-1}{l-1}\Leftrightarrow x_{1,\ldots ,l}=1+\left(l-1\right)\cdot r_{1,\ldots ,l},$$

and is demonstrated in Figure 3.

Then $x_{1,\ldots ,l}$ are pseudo-random numbers uniformly distributed in the range from 1 to l.

Thus, we can form a set of non-repeating numbers

(11) $$Key1=\left\{x_{1},x_{2},\ldots ,x_{l}\right\},$$

which will correspond to the new indexes of each character of text information $T_{1,\ldots ,l}$. This set of numbers Key1 will correspond to Key 1, which is used at the stage of integrating text into an audio signal (Figure 1) and at the stage of extracting text from an audio signal (Figure 2).

Then, the operations of interleaving, which is used at the stage of integrating text into an audio signal (Figure 1), and de-interleaving, which is used at the stage of extracting text from an audio signal (Figure 2), can be represented as follows:

(12) $$T_{Key1}=T_{1,\ldots ,l}\left(Key1_{1,\ldots ,l}\right),$$

(13) $$T_{1,\ldots ,l}=T_{Key1}\left(Key1_{1,\ldots ,l}\right).$$

Since the low-frequency wavelet coefficients will increase their absolute power with each next level of decomposition, then the text information $T_{Key1}$ must be sorted in such a way that its integration into low-frequency wavelet coefficients occurs from the minimum min to the maximum max values in accordance with the expression $\left\{\min\left(T_{Key1}\right),\ldots ,\max\left(T_{Key1}\right)\right\}$; this is the main task of applying the sorting operation.

So, having received text information $T_{Key1}$ that was subject to the interleaving operation using Key1, it is necessary to perform a sorting operation from the minimum min to the maximum max value of the set of characters $T_{Key1}$.

We presented the input text information in the form of a set $T=\left\{S_{i},S_{i},\ldots ,S_{i}\right\}$, where $S=\left\{0,1,2,\ldots ,127\right\}$ is a set of numbers that correspond to each specific character according to the ASCII encoding, and i is determined by the initial sequence of characters in the text. Therefore, the expression can be rewritten as $T_{Key1}=\left\{S_{i},S_{i},\ldots ,S_{i}\right\}$, where i defines a sequence of numbers in the range from 0 to 127 depending on Key1.

Then, the operations of sorting, which is used at the stage of integrating text into an audio signal (Figure 1), and de-sorting, which is used at the stage of extracting text from an audio signal (Figure 2), can be written as follows:

(14) $$T_{Key2}=T_{Key1}\left(Key2_{1,\ldots ,l}\right),$$

(15) $$T_{Key1}=T_{Key2}\left(Key2_{1,\ldots ,l}\right),$$

where $Key2_{1,\ldots ,l}$ is the sequence of indexes of the set of characters $T_{Key1}$ that was formed according to the expression $\left\{\min\left(T_{Key1}\right),\ldots ,\max\left(T_{Key1}\right)\right\}$, which corresponds to Key 2 in Figure 1 and Figure 2.

So, having text information $T_{Key2}$ that has undergone a sorting operation according to the condition $\left\{\min\left(T_{Key1}\right),\ldots ,\max\left(T_{Key1}\right)\right\}$, it needs to be divided into L−1 blocks, where L is a maximum number of levels of wavelet decomposition of the audio signal, since there is no text integration at the last level of wavelet decomposition (Figure 1).

Then, the number of blocks b of text information $T_{Key2}$ is determined by finding the maximum level of wavelet decomposition L of the audio signal, which can be expressed as follows:

(16) b=L−1,

(17) $$L\approx \log_{2}\left(\frac{l_{A}}{l_{F}-1}\right).$$

The correctness of this expression is confirmed by the fulfillment of the condition

(18) $$\left(l_{F}-1\right)\cdot 2^{L}<l_{A},$$

where $l_{A}$ is a number of samples of the audio signal, $l_{F}$ is a number of coefficients of the Daubechies wavelet filter, and the symbol ≈ characterizes the rounding down of a number L [27,28,29].

Then, the number of characters in one block $l_{b}$ of text information $T_{Key2}$ is determined according to the expression

(19) $$l_{b}=\frac{l_{T}}{b},$$

where $l_{T}$ is a total number of characters of text information $T_{Key2}$ that should be hidden in the audio signal.

It should be noted that the number of characters of text information in one block $l_{b}$ directly depends on the maximum level of wavelet decomposition L of the audio signal, as can be seen from Formulas (16)–(19). Then, finding the maximum level of wavelet decomposition L allows for uniformly integrating all blocks b of text information $T_{Key2}$ at all decomposition levels 1,…,L−1 to increase the resistance to audio signal compression, since with an increase in the decomposition level, the amplitude of the wavelet coefficients will increase and, accordingly, the amplitude of the text information integrated into them, due to the subsequent scalar product with a wavelet filter at each decomposition level 1,…,L−1, which is a characteristic feature of the proposed method.

Thus text information $T_{Key2}$, which is divided into b blocks, where the number of characters in one block is $l_{b}$, can be represented in the form of a set

(20) $$Tb_{1,\ldots ,b}=\left\{T_{1,\ldots ,l_{b}},T_{l_{b}+1,\ldots ,2l_{b}},T_{2l_{b}+1,\ldots ,3l_{b}},\ldots ,T_{\left(b-1\right)l_{b}+1,\ldots ,bl_{b}}\right\},$$

where $T=T_{Key2}$, $Tb_{1}=T_{1,\ldots ,l_{b}}$, $Tb_{2}=T_{l_{b}+1,\ldots ,2l_{b}}$, $Tb_{3}=T_{2l_{b}+1,\ldots ,3l_{b}}$, $Tb_{b}=T_{\left(b-1\right)l_{b}+1,\ldots ,bl_{b}}$, which corresponds to the operation of dividing text information into blocks, which is used at the stage of integrating text into an audio signal, according to Figure 1.

Then, the operation of combining blocks of text information $Tb_{1,\ldots ,b}$, which is used at the stage of extracting text from an audio signal, according to Figure 2, will look like this:

(21) $$T_{Key2}=\overset{b}{\underset{i=1}{\cup }}Tb_{i}.$$

At the final stage of preparing text information for integration into an audio signal, it is necessary to perform the normalization operation

(22) $$Tbn_{1,\ldots ,b}=\frac{Tb_{1,\ldots ,b}}{\max\left(Tb_{1,\ldots ,b}\right)},$$

(23) $$An_{1,\ldots ,l_{A}}=\frac{A_{1,\ldots ,l_{A}}}{\max\left(A_{1,\ldots ,l_{A}}\right)},$$

so that text information $Tbn_{1,\ldots ,b}$ and audio signal $An_{1,\ldots ,l_{A}}$ are in the same normalization scale, namely, so that values of ASCII codes of text characters $Tb_{1,\ldots ,b}$ and audio signal samples $A_{1,\ldots ,l_{A}}$ are in the range from 0 to 1.

Then, the restoration of the normalized text information $Tbn_{1,\ldots ,b}$ and the audio signal $An_{1,\ldots ,l_{A}}$ to the original normalization (de-normalization) scale can be carried out according to the expressions

(24) $$Tb_{1,\ldots ,b}=Tbn_{1,\ldots ,b}\cdot \max\left(Tb_{1,\ldots ,b}\right),$$

(25) $$A_{1,\ldots ,l_{A}}=An_{1,\ldots ,l_{A}}\cdot \max\left(A_{1,\ldots ,l_{A}}\right),$$

where this sequence of operations corresponds to the blocks of normalization and de-normalization, which are used at the stage of integrating text into an audio signal (Figure 1) and extracting text from an audio signal (Figure 2).

Thus, we get blocks of normalized text information $Tbn_{1,\ldots ,b}$ that are ready for integration into a normalized audio signal $An_{1,\ldots ,l_{A}}$. However, since the integration does not take place in the audio signal $An_{1,\ldots ,l_{A}}$ itself, but in its low-frequency wavelet coefficients (LFWC) followed by their scalar product with low-frequency (LPF-D) and high-frequency (HPF-D) orthogonal Daubechies wavelet filters at each 1,…,L−1 level of the wavelet decomposition, it is necessary to perform a wavelet transform of the audio signal $An_{1,\ldots ,l_{A}}$ and find the low-frequency (LFWC) and high-frequency (HFWC) wavelet coefficients for each 1,…,L level of the wavelet decomposition [30,31]. It should be noted that not only Daubechies filters can be used, but also other orthogonal wavelet filters, such as Coiflets, Symlets, or Meyer.

Then, the discrete wavelet transform is the scalar product of the values of the studied audio signal $An_{1,\ldots ,l_{A}}$, with the coefficients of the orthogonal Daubechies wavelet filters of low D (LPF-D) and high V (HPF-D) frequencies for decomposition, followed by a double thinning ↓2 of the obtained coefficients

(26) $$Z{\left(1\right)}_{1,\ldots ,K}↓2={\left\{Z{\left(1\right)}_{2},Z{\left(1\right)}_{4},Z{\left(1\right)}_{6},\ldots ,Z{\left(1\right)}_{K}\right\}}_{1,\ldots ,K/2},$$

(27) $$Y{\left(1\right)}_{1,\ldots ,K}↓2={\left\{Y{\left(1\right)}_{2},Y{\left(1\right)}_{4},Y{\left(1\right)}_{6},\ldots ,Y{\left(1\right)}_{K}\right\}}_{1,\ldots ,K/2},$$

which can be formalized as follows:

(28) $$Z{\left(1\right)}_{1,\ldots ,K/2}={\left(\sum_{j=\max\left(1,\;k+1-l_{F}\right)}^{\min\left(k,\;l_{A}\right)}{\left(An_{1,\ldots ,l_{A}}\right)}_{j}D_{i}\right)}_{1,\ldots ,K}↓2,$$

(29) $$Y{\left(1\right)}_{1,\ldots ,K/2}={\left(\sum_{j=\max\left(1,\;k+1-l_{F}\right)}^{\min\left(k,\;l_{A}\right)}{\left(An_{1,\ldots ,l_{A}}\right)}_{j}V_{i}\right)}_{1,\ldots ,K}↓2,$$

where $K=l_{A}+l_{F}-1$, k=1,…,K, i=k+1−j, and $Z{\left(1\right)}_{1,\ldots ,K/2}$, $Y{\left(1\right)}_{1,\ldots ,K/2}$ are low-frequency (LFWC) and high-frequency (HFWC) wavelet coefficients for the 1st level of audio signal $An_{1,\ldots ,l_{A}}$ decomposition [32,33].

Since the text information $Tbn_{1,\ldots ,b}$ has been sorted from minimum min to maximum max values according to the expression $\left\{\min\left(T_{Key1}\right),\ldots ,\max\left(T_{Key1}\right)\right\}$, to find the indexes of values (Key 3) of low-frequency wavelet coefficients $Z{\left(1\right)}_{1,\ldots ,K/2}$, which should be replaced → with the corresponding block of text information $Tbn_{1}$, it is also necessary to sort the low-frequency wavelet coefficients $Z{\left(1\right)}_{1,\ldots ,K/2}$ from the minimum min to the maximum max values according to the expression $\left\{\min\left(\left|Z{\left(1\right)}_{1,\ldots ,K/2}\right|\right),\ldots ,\max\left(\left|Z{\left(1\right)}_{1,\ldots ,K/2}\right|\right)\right\}$ and determine the indexes $Key3_{1,\ldots ,l_{b}}$ of absolute minimum values $1,\ldots ,l_{b}$, which can be written as follows:

(30) $$Key3_{1,\ldots ,l_{b}}={\left\{\min\left(\left|Z{\left(1\right)}_{1,\ldots ,K/2}\right|\right),\ldots ,\max\left(\left|Z{\left(1\right)}_{1,\ldots ,K/2}\right|\right)\right\}}_{1,\ldots ,l_{b}},$$

where $l_{b}$ is the number of characters in one block of text information $Tbn_{1,\ldots ,b}$.

Then, the operations of integrating → text information $Tbn_{1}$ into low-frequency wavelet coefficients $Z{\left(1\right)}_{1,\ldots ,K/2}$ (Figure 1) and extracting text information $Tbn_{1}$ from low-frequency wavelet coefficients $Z{\left(1\right)}_{Tbn_{1}}$ (Figure 2) can be written as follows

(31) $$Z{\left(1\right)}_{Tbn_{1}}=Tbn_{1}\to Z{\left(1\right)}_{1,\ldots ,K/2}\left(Key3_{1,\ldots ,l_{b}}\right),$$

(32) $$Tbn_{1}=Z{\left(1\right)}_{Tbn_{1}}\left(Key3_{1,\ldots ,l_{b}}\right),$$

where $Key3_{1,\ldots ,l_{b}}$ is a sequence of indexes of the absolute minimum values of low-frequency wavelet coefficients $Z{\left(1\right)}_{1,\ldots ,K/2}$, which was formed according to the condition ${\left\{\min\left(\left|Z{\left(1\right)}_{1,\ldots ,K/2}\right|\right),\ldots ,\max\left(\left|Z{\left(1\right)}_{1,\ldots ,K/2}\right|\right)\right\}}_{1,\ldots ,l_{b}}$, and corresponds to Key 3 in Figure 1 and Figure 2.

This operation is needed in order to replace → the absolute minimum values of low-frequency wavelet coefficients $Z{\left(1\right)}_{1,\ldots ,l_{b}}$ with the minimum values of text information $Tbn_{1,\ldots ,l_{b}}$, which can be formalized by the following relation:

(33) $$Z{\left(1\right)}_{Tbn_{1}}=\left\{Tbn_{1,\ldots ,l_{b}}\to Z{\left(1\right)}_{1,\ldots ,l_{b}},Z{\left(1\right)}_{l_{b}+1},\ldots ,Z{\left(1\right)}_{K/2}\right\},$$

where $Z{\left(1\right)}_{1,\ldots ,K/2}={\left\{\min\left(\left|Z{\left(1\right)}_{1,\ldots ,K/2}\right|\right),\ldots ,\max\left(\left|Z{\left(1\right)}_{1,\ldots ,K/2}\right|\right)\right\}}_{1,\ldots ,K/2}$.

This approach will provide less distortion of the audio signal $An_{1,\ldots ,l_{A}}$ during its inverse recovery $A{n^{\prime }}_{1,\ldots ,l_{A}}$ by wavelet coefficients $Z{\left(1\right)}_{1,\ldots ,K/2}$ and $Y{\left(1\right)}_{1,\ldots ,K/2}$, since both the audio signal $An_{1,\ldots ,l_{A}}$ and text information $Tbn_{1,\ldots ,l_{b}}$ are in the same normalization scale, namely from 0 to 1, which allows us to correlate their absolute power [34,35].

Then, the operation of recursive integrating → of all blocks of text information $Tbn_{1,\ldots ,b}$ into low-frequency wavelet coefficients $Z{\left(1,\ldots ,L-1\right)}_{1,\ldots ,K/2}$ at all 1,…,L−1 levels of the wavelet decomposition of the audio signal $An_{1,\ldots ,l_{A}}$ followed by their scalar product with low-frequency $D_{i}$ (LPF-D) and high-frequency $V_{i}$ (HPF-D) orthogonal Daubechies wavelet filters for decomposition (Figure 1) can be written as follows:

(34) $$Z{\left(1\right)}_{1,\ldots ,K/2}={\left(\sum_{j=\max\left(1,\;k+1-l_{F}\right)}^{\min\left(k,\;l_{A}\right)}{\left(An_{1,\ldots ,l_{A}}\right)}_{j}D_{i}\right)}_{1,\ldots ,K}↓2$$

(35) $$Y{\left(1\right)}_{1,\ldots ,K/2}={\left(\sum_{j=\max\left(1,\;k+1-l_{F}\right)}^{\min\left(k,\;l_{A}\right)}{\left(An_{1,\ldots ,l_{A}}\right)}_{j}V_{i}\right)}_{1,\ldots ,K}↓2$$

(36) $$Z{\left(1\right)}_{Tbn_{1}}=Tbn_{1}\to Z{\left(1\right)}_{1,\ldots ,K/2}\left(Key3_{1}\right)$$

where $K=l_{A}+l_{F}-1$, k=1,…,K, i=k+1−j, $Key3_{1}={\left\{\min\left(\left|Z{\left(1\right)}_{1,\ldots ,K/2}\right|\right),\ldots ,\max\left(\left|Z{\left(1\right)}_{1,\ldots ,K/2}\right|\right)\right\}}_{1,\ldots ,l_{b}}$, and

(37) $$Z{\left(2\right)}_{1,\ldots ,K/2}={\left(\sum_{j=\max\left(1,\;k+1-l_{F}\right)}^{\min\left(k,\;l_{Z\left(1\right)}\right)}{\left(Z{\left(1\right)}_{Tbn_{1}}\right)}_{j}D_{i}\right)}_{1,\ldots ,K}↓2,$$

(38) $$Y{\left(2\right)}_{1,\ldots ,K/2}={\left(\sum_{j=\max\left(1,\;k+1-l_{F}\right)}^{\min\left(k,\;l_{Z\left(1\right)}\right)}{\left(Z{\left(1\right)}_{Tbn_{1}}\right)}_{j}V_{i}\right)}_{1,\ldots ,K}↓2,$$

(39) $$Z{\left(2\right)}_{Tbn_{2}}=Tbn_{2}\to Z{\left(2\right)}_{1,\ldots ,K/2}\left(Key3_{2}\right),$$

where $K=l_{Z\left(1\right)}+l_{F}-1$, k=1,…,K, i=k+1−j, $Key3_{2}={\left\{\min\left(\left|Z{\left(2\right)}_{1,\ldots ,K/2}\right|\right),\ldots ,\max\left(\left|Z{\left(2\right)}_{1,\ldots ,K/2}\right|\right)\right\}}_{1,\ldots ,l_{b}}$, and

(40) $$Z{\left(L-1\right)}_{1,\ldots ,K/2}={\left(\sum_{j=\max\left(1,\;k+1-l_{F}\right)}^{\min\left(k,\;l_{Z\left(2\right)}\right)}{\left(Z{\left(2\right)}_{Tbn_{2}}\right)}_{j}D_{i}\right)}_{1,\ldots ,K}↓2,$$

(41) $$Y{\left(L-1\right)}_{1,\ldots ,K/2}={\left(\sum_{j=\max\left(1,\;k+1-l_{F}\right)}^{\min\left(k,\;l_{Z\left(2\right)}\right)}{\left(Z{\left(2\right)}_{Tbn_{2}}\right)}_{j}V_{i}\right)}_{1,\ldots ,K}↓2,$$

(42) $$Z{\left(L-1\right)}_{Tbn_{b}}=Tbn_{b}\to Z{\left(L-1\right)}_{1,\ldots ,K/2}\left(Key3_{b}\right),$$

where $K=l_{Z\left(2\right)}+l_{F}-1$, k=1,…,K, i=k+1−j, $Key3_{b}={\left\{\min\left(\left|Z{\left(L-1\right)}_{1,\ldots ,K/2}\right|\right),\ldots ,\max\left(\left|Z{\left(L-1\right)}_{1,\ldots ,K/2}\right|\right)\right\}}_{1,\ldots ,l_{b}}$, and

(43) $$Z{\left(L\right)}_{1,\ldots ,K/2}={\left(\sum_{j=\max\left(1,\;k+1-l_{F}\right)}^{\min\left(k,\;l_{Z\left(L-1\right)}\right)}{\left(Z{\left(L-1\right)}_{Tbn_{b}}\right)}_{j}D_{i}\right)}_{1,\ldots ,K}↓2,$$

(44) $$Y{\left(L\right)}_{1,\ldots ,K/2}={\left(\sum_{j=\max\left(1,\;k+1-l_{F}\right)}^{\min\left(k,\;l_{Z\left(L-1\right)}\right)}{\left(Z{\left(L-1\right)}_{Tbn_{b}}\right)}_{j}V_{i}\right)}_{1,\ldots ,K}↓2,$$

where $K=l_{Z\left(L-1\right)}+l_{F}-1$, k=1,…,K, i=k+1−j, and $Z{\left(1,\ldots ,L\right)}_{1,\ldots ,K/2}$, $Y{\left(1,\ldots ,L\right)}_{1,\ldots ,K/2}$ are low-frequency and high-frequency wavelet coefficients for 1,…,L, the levels of audio signal $An_{1,\ldots ,l_{A}}$ and decomposition $Z{\left(1,\ldots ,L-1\right)}_{Tbn_{1,\ldots ,b}}$ are low-frequency wavelet coefficients of decomposition levels 1,…,L−1 with integrated → blocks of text information $Tbn_{1,\ldots ,b}$ in accordance with $Key3_{1,\ldots ,b}={\left\{Key3_{1},Key3_{2},\ldots ,Key3_{b}\right\}}_{1,\ldots ,bl_{b}}$.

If we shorten expressions (34)–(44), we obtain the operation of recursive integrating → of text information $Tbn_{1,\ldots ,b}$ into low-frequency wavelet coefficients $Z{\left(1,\ldots ,L-1\right)}_{1,\ldots ,K/2}$ of the audio signal $An_{1,\ldots ,l_{A}}$ (Figure 1), according to the following formulas:

(45) $$Z{\left(1\right)}_{1,\ldots ,K/2}={\left(\sum_{j=\max\left(1,\;k+1-l_{F}\right)}^{\min\left(k,\;l_{A}\right)}{\left(An_{1,\ldots ,l_{A}}\right)}_{j}D_{i}\right)}_{1,\ldots ,K}↓2,$$

(46) $$Y{\left(1\right)}_{1,\ldots ,K/2}={\left(\sum_{j=\max\left(1,\;k+1-l_{F}\right)}^{\min\left(k,\;l_{A}\right)}{\left(An_{1,\ldots ,l_{A}}\right)}_{j}V_{i}\right)}_{1,\ldots ,K}↓2,$$

where $K=l_{A}+l_{F}-1$, k=1,…,K, i=k+1−j;

(47) $$Z{\left(1,\ldots ,L-1\right)}_{Tbn_{1,\ldots ,b}}=Tbn_{1,\ldots ,b}\to Z{\left(1,\ldots ,L-1\right)}_{1,\ldots ,K/2}\left(Key3_{1,\ldots ,b}\right),$$

(48) $$Z{\left(2,\ldots ,L\right)}_{1,\ldots ,K/2}={\left(\sum_{j=\max\left(1,\;k+1-l_{F}\right)}^{\min\left(k,\;l_{Z\left(1,\ldots ,L-1\right)}\right)}{\left(Z{\left(1,\ldots ,L-1\right)}_{Tbn_{1,\ldots ,b}}\right)}_{j}D_{i}\right)}_{1,\ldots ,K}↓2,$$

(49) $$Y{\left(2,\ldots ,L\right)}_{1,\ldots ,K/2}={\left(\sum_{j=\max\left(1,\;k+1-l_{F}\right)}^{\min\left(k,\;l_{Z\left(1,\ldots ,L-1\right)}\right)}{\left(Z{\left(1,\ldots ,L-1\right)}_{Tbn_{1,\ldots ,b}}\right)}_{j}V_{i}\right)}_{1,\ldots ,K}↓2,$$

where $K=l_{Z\left(1,\ldots ,L-1\right)}+l_{F}-1$, k=1,…,K, i=k+1−j, $Key3_{1,\ldots ,b}={\left\{\min\left(\left|Z{\left(1,\ldots ,L-1\right)}_{1,\ldots ,K/2}\right|\right),\ldots ,\max\left(\left|Z{\left(1,\ldots ,L-1\right)}_{1,\ldots ,K/2}\right|\right)\right\}}_{1,\ldots ,bl_{b}}$.

Then, to reconstruct the audio signal $A{n^{\prime }}_{1,\ldots ,l_{A}}$ with the text $Tbn_{1,\ldots ,b}$ integrated → into it (Figure 1), it is required to perform the operation of doubling ↑2 the low-frequency $Z{\left(1,\ldots ,L-1\right)}_{Tbn_{1,\ldots ,b}}$, $Z{\left(L\right)}_{1,\ldots ,K/2}$

(50) $$Z{\left(1,\ldots ,L-1\right)}_{Tbn_{1,\ldots ,b}}↑2={\left\{\begin{array}{l} {\left(Z{\left(1,\ldots ,L-1\right)}_{Tbn_{1,\ldots ,b}}\right)}_{1},0,{\left(Z{\left(1,\ldots ,L-1\right)}_{Tbn_{1,\ldots ,b}}\right)}_{2},0,\ldots \\ \ldots ,0,{\left(Z{\left(1,\ldots ,L-1\right)}_{Tbn_{1,\ldots ,b}}\right)}_{K/2} \end{array}\right\}}_{1,\ldots ,K},$$

(51) $$Z{\left(L\right)}_{1,\ldots ,K/2}↑2={\left\{Z{\left(L\right)}_{1},0,Z{\left(L\right)}_{2},0,\ldots ,0,Z{\left(L\right)}_{K/2}\right\}}_{1,\ldots ,K},$$

and high-frequency $Y{\left(1,\ldots ,L\right)}_{1,\ldots ,K/2}$

(52) $$Y{\left(1,\ldots ,L\right)}_{1,\ldots ,K/2}↑2={\left\{Y{\left(1,\ldots ,L\right)}_{1},0,Y{\left(1,\ldots ,L\right)}_{2},0,\ldots ,0,Y{\left(1,\ldots ,L\right)}_{K/2}\right\}}_{1,\ldots ,K},$$

wavelet coefficients followed by the sum of the results of their scalar products with the coefficients of the orthogonal Daubechies wavelet filters of low R (LPF-R) and high W (HPF-R) frequencies for reconstruction at each 1,…,L level of the wavelet decomposition, according to the expression

(53) $$\begin{array}{ll} An_{1,\ldots ,l_{A}}^{'} & =\left({\left(\overset{\min\left(k,2l_{Z\left(1,\ldots ,L-1\right)}-1\right)}{\underset{j=\max\left(1,\;k+1-l_{F}\right)}{\sum }}{\left(Z{\left(1,\ldots ,L-1\right)}_{Tbn_{1,\ldots ,b}}↑2\right)}_{j}R_{i}\right)}_{1,\ldots ,K}+{\left(\overset{\min\left(k,2l_{Z\left(1,\ldots ,L-1\right)}-1\right)}{\underset{j=\max\left(1,\;k+1-l_{F}\right)}{\sum }}{\left(Y{\left(1,\ldots ,L-1\right)}_{1,\ldots ,\frac{K}{2}}↑2\right)}_{j}W_{i}\right)}_{1,\ldots ,K}\right)+\\ & +\left({\left(\overset{\min\left(k,2l_{Z\left(L\right)}-1\right)}{\underset{j=\max\left(1,\;k+1-l_{F}\right)}{\sum }}{\left(Z{\left(L\right)}_{1,\ldots ,\frac{K}{2}}↑2\right)}_{j}R_{i}\right)}_{1,\ldots ,K}+{\left(\overset{\min\left(k,2l_{Z\left(L\right)}-1\right)}{\underset{j=\max\left(1,\;k+1-l_{F}\right)}{\sum }}{\left(Y{\left(L\right)}_{1,\ldots ,\frac{K}{2}}↑2\right)}_{j}W_{i}\right)}_{1,\ldots ,K}\right), \end{array}$$

where $K=2l_{Z\left(1,\ldots ,L\right)}-1+l_{F}-1$, k=1,…,K, i=k+1−j.

Then, the operation of recursively extracting all blocks of text information $Tbn_{1,\ldots ,b}$ from low-frequency wavelet coefficients $Z{\left(1,\ldots ,L-1\right)}_{Tbn_{1,\ldots ,b}}$ at all 1,…,L−1 levels of the wavelet decomposition of the audio signal $A{n^{\prime }}_{1,\ldots ,l_{A}}$ (Figure 2) can be represented as follows:

(54) $$Z{\left(1\right)}_{Tbn_{1}}={\left(\sum_{j=\max\left(1,\;k+1-l_{F}\right)}^{\min\left(k,\;l_{A}\right)}{\left(A{n^{\prime }}_{1,\ldots ,l_{A}}\right)}_{j}D_{i}\right)}_{1,\ldots ,K}↓2,$$

where $K=l_{A}+l_{F}-1$, k=1,…,K, i=k+1−j,

(55) $$Tbn_{1,\ldots ,b}=Z{\left(1,\ldots ,L-1\right)}_{Tbn_{1,\ldots ,b}}\left(Key3_{1,\ldots ,b}\right),$$

(56) $$Z{\left(2,\ldots ,L-1\right)}_{Tbn_{2,\ldots ,b}}={\left(\sum_{j=\max\left(1,\;k+1-l_{F}\right)}^{\min\left(k,\;l_{Z\left(1,\ldots ,L-2\right)}\right)}{\left(Z{\left(1,\ldots ,L-2\right)}_{Tbn_{1,\ldots ,b-1}}\right)}_{j}D_{i}\right)}_{1,\ldots ,K}↓2,$$

where $K=l_{Z\left(1,\ldots ,L-2\right)}+l_{F}-1$, k=1,…,K, i=k+1−j, $Key3_{1,\ldots ,b}={\left\{Key3_{1},Key3_{2},\ldots ,Key3_{b}\right\}}_{1,\ldots ,bl_{b}}$.

Thus, we have the following operations:

(1) integrating → text information $Tbn_{1,\ldots ,b}$ into low-frequency wavelet coefficients $Z{\left(1,\ldots ,L-1\right)}_{1,\ldots ,K/2}$ of an audio signal $An_{1,\ldots ,l_{A}}$ followed by their scalar product with low-frequency $D_{i}$ (LPF-D) and high-frequency $V_{i}$ (HPF-D) orthogonal Daubechies wavelet filters for decomposition (45)–(49) (Listing A1 in Appendix A);

(2) reconstructing the audio signal $A{n^{\prime }}_{1,\ldots ,l_{A}}$ with the text $Tbn_{1,\ldots ,b}$ integrated → into it by low-frequency $Z{\left(1,\ldots ,L-1\right)}_{Tbn_{1,\ldots ,b}}$, $Z{\left(L\right)}_{1,\ldots ,K/2}$ and high-frequency $Y{\left(1,\ldots ,L\right)}_{1,\ldots ,K/2}$ wavelet coefficients (53) (Listing A2 in Appendix A);

(3) extracting text information $Tbn_{1,\ldots ,b}$ from low-frequency wavelet coefficients $Z{\left(1,\ldots ,L-1\right)}_{Tbn_{1,\ldots ,b}}$ of the audio signal $A{n^{\prime }}_{1,\ldots ,l_{A}}$ (54)–(56) (Listing A3 in Appendix A).

These are the main scientific results of the proposed method of steganographic hiding of text information in an audio signal based on the wavelet transform.

## 3. Results of Scientific Experimental Research

A computer model of the method of steganographic protection of text information based on the wavelet transform was modeled and studied in the MATLAB R2021b software and mathematical complex using a set of the following libraries: Signal Processing Toolbox, Wavelet Toolbox, Audio Toolbox, Text Analytics Toolbox, Filter Design HDL Coder, DSP System Toolbox, Communications Toolbox, Statistics and Machine Learning Toolbox.

In the experimental study, the initial audio signal for the proposed method of steganographic hiding of text information is a mono recording of the announcer in a male voice. The duration of mono recording is 91 s of the poem *The Road Not Taken*, by Robert Frost, in audio format WAV with a sampling rate of 44.1 kHz and a quantization bit depth of 16 bits per sample. Therefore, the stream of the bit sequence of audio data at the input of the computer model of the developed method will be—705.6 Kbps, and the total amount of audio data will be—7.8 MB.

The audio signal was recorded using a sound card with a maximum sampling rate of 192 kHz, number of bits per sample of 24 bits/sample, and a signal-to-noise ratio of 116 dB using a unidirectional 16-bit condenser microphone with an audio sensitivity of 110 dB.

**Figure 4:**
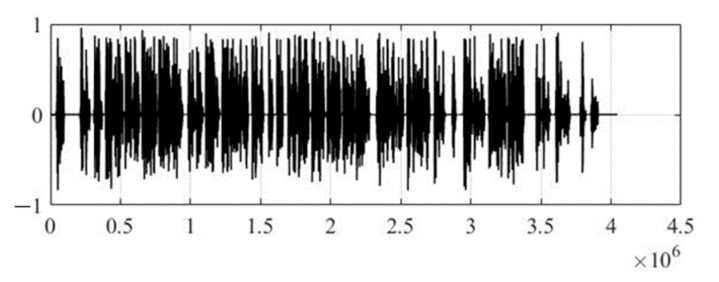
Original audio signal.

**Figure 5:**
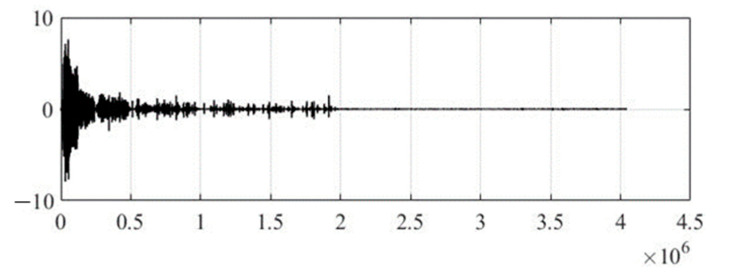
Wavelet coefficients of the original audio signal.

Figure 4 shows the original audio signal before steganographic processing to embed secret text information, and Figure 5 shows the wavelet coefficients of the 17th level of decomposition, where the Daubechies function of the 12th order was used as a generating wavelet function. It should be noted that the optimal choice of the generating wavelet function and the number of decomposition levels are not trivial tasks, since the speech signal is a non-stationary process, and it is not possible to predict changes in its spectral component over time. Therefore, in practice, it is recommended to use the smoothest wavelet functions with a large number of zero moments (function order) and maximum number of possible levels of decomposition, which is determined through the energy of the signal under study and the wavelet function. This will make the wavelet spectrum of the speech signal most suitable for integrating text information.

As the initial text information (to be hidden) in the method under study, the poem *The Road Not Taken* by Robert Frost was used in text format TXT in the amount of 740 characters according to the rules of ASCII encoding, where 8 bits are allocated per character, from which it follows that the total amount of text information at the input of the computer model of the developed method will be 740 bytes.

**Figure 6:**
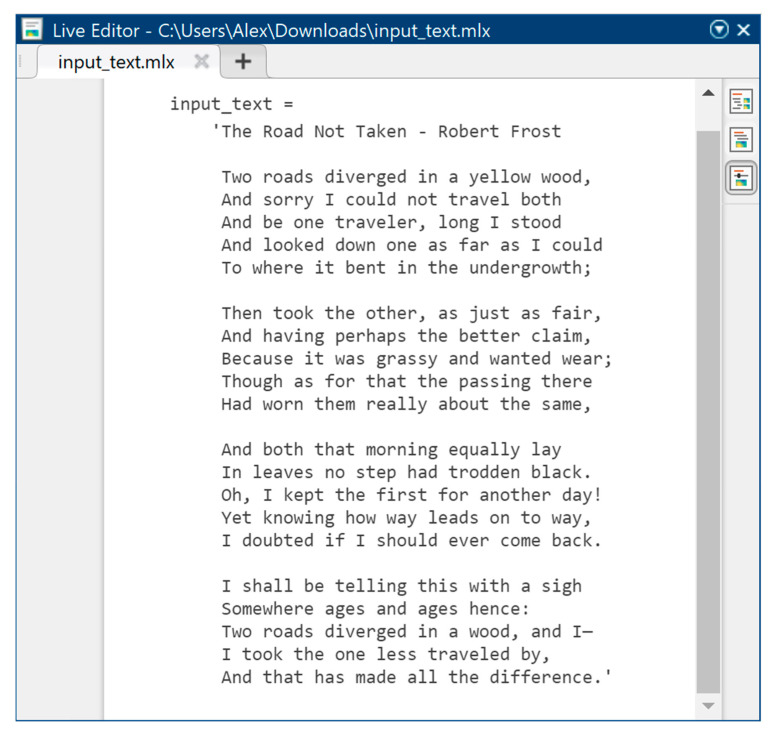
Original text information in the form of characters.

**Figure 7:**
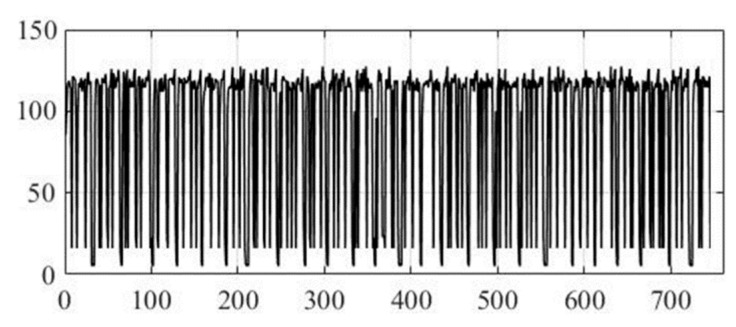
Original text information in the form of ASCII codes.

Figure 6 shows the original text information in symbolic form before steganographic embedding in order to hide it in the audio signal, taking into account the psychophysiological features of human hearing. Figure 7 also shows text information, but already encoded according to the ASCII encoding rules. It is the normalized values of ASCII codes that we must mask as best as possible in a highly redundant audio data stream, to hide the very fact of text transmission.

Table 1 presents the results of an experimental study, namely, quantitative estimates of the effectiveness of the existing stego-system based on wavelet transform under conditions of passive or deliberate distortion of text information hidden in the audio signal were obtained by applying redundancy reduction methods (compression).

The main task formulated earlier is to increase the robustness of the stego-system to compression algorithms, so that when compressing a steganocoded audio signal, the text information that is hidden inside it remains as complete as possible. Objective metrics are used to automate the processes of evaluating the effectiveness of embedding text information in an audio signal, which allow evaluating the distortions introduced by the stego-system into the original audio signal. As such, criteria for evaluating the effectiveness of the stego-system include objective metrics such as compression ratio (CR), correlation coefficient (CC), normalized root mean square error (NRMSE), and signal-to-noise ratio (SNR), peak signal-to-noise ratio (PSNR). It should be noted that CC, NRMSE, SNR, and PSNR are very sensitive to changes in the amplitude of the audio signal. Since it is the change in the amplitude of the audio signal that characterizes the degree of its distortion, this is exactly what we need to evaluate the quality of masking text information in an audio container, since this process entails signal distortion (amplitude distortion). Also, in this experimental study, Daubechies wavelet filters of the 12th order were used. This fact should be taken into account when interpreting the results obtained in CR, CC, NRMSE, SNR, and PSNR, which directly depend on the specific implementation of the audio signal, text information, and the selected wavelet filter, which will result in changes in the critical compression threshold in different versions of the experiment.

The obtained values of performance indicators should be interpreted as follows: with CR = 1, the steganocoded audio signal is not subjected to distortions introduced by the compression algorithm; at the same time, a very high psychophysiological model of sound perception (masking) is observed, which is confirmed by indicators CC = 0.9999, NRMSE = 0.0060, SNR = 36.7794, and PSNR = 63.0616. In this case, text information, when extracted from the audio signal, has ideal performance CC = 1, NRMSE = 0, SNR = ∞, and PSNR = ∞, and this means that text information has not been subjected to the slightest distortion and is completely integral. The infinity symbol ∞ in this case means an infinitely high value of the criterion. According to Table 1, the parsed text information will match the full copy of the input text; that is, at the output of the transformations, we will have a text of the form as in Figure 6.

**Figure 8:**
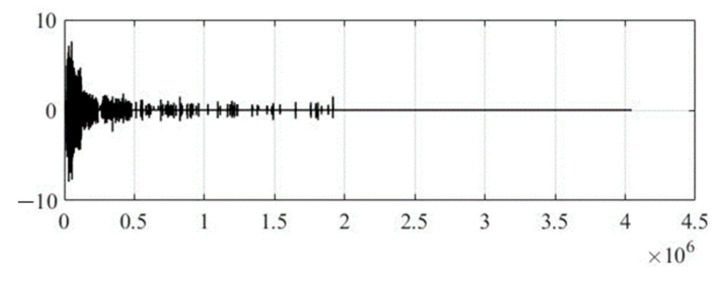
Wavelet coefficients of compressed steganocoded audio signal by six times with full integrity of text information (existing method).

Figure 8 shows the wavelet coefficients after the audio signal is compressed by six times, but the integrity of the text information remains unchanged, which is ideal.

It should be noted that in the existing method, the indicator CR = 6 is the boundary value at which text information is not subjected to distortions of the compression algorithm; this can be seen by analyzing the values CC = 1, NRMSE = 0, SNR = ∞, and PSNR = ∞ while maintaining a sufficient quality indicator in terms of masking according to CC = 0.9861, NRMSE = 0.0681, SNR = 30.2330, and PSNR = 51.6068. In other words, at a compression level of six times, there are no audible differences between the original and steganocoded audio signals. This is the so-called ‘critical level of compression’, at which there is no distortion of text information, by raising the threshold above the critical compression level, distortion occurs.

**Figure 9:**
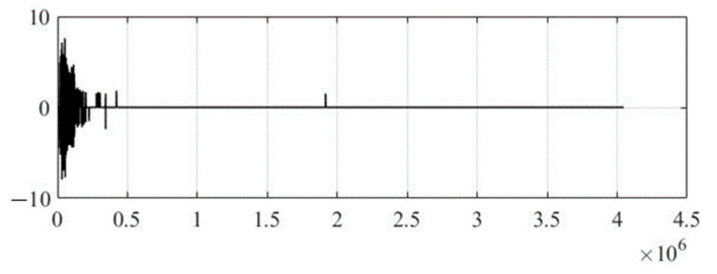
Wavelet coefficients of compressed steganocoded audio signal by 30 times (existing method).

For clarity, we present the values of the wavelet coefficients of the compressed steganocoded audio signal by a factor of 30 in Figure 9. From CC = 0.8632, NRMSE = 0.4984, SNR = 6.8333, and PSNR = 11.8327, it can be seen that under such conditions, it is not necessary to talk about the good sound quality of the audio signal. Also, due to the fact that there is a significant reduction in the redundancy of the steganocoded audio signal, it becomes problematic to maintain the integrity of text information in it.

**Figure 10:**
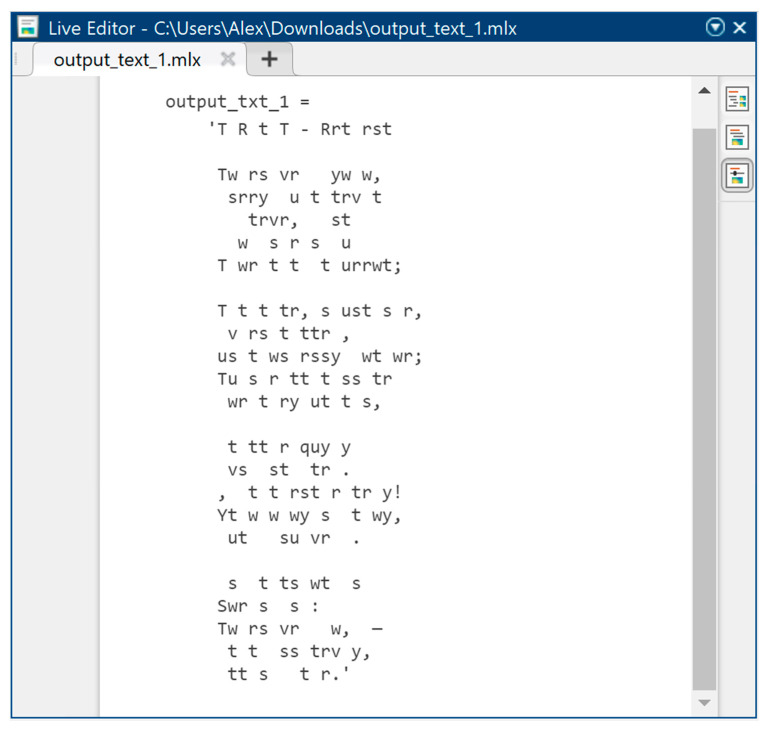
Recovered text information after 30-times compression of steganocoded audio signal (existing method).

Consider what happens to text information with such compression. Figure 10 shows the recovered text information when the steganocoded audio signal is compressed by 30 times. According to the indicators from Table 1, with CR = 30, we have text distortion in proportion to the values CC = 0.7453, NRMSE = 0.6893, SNR = 4.0383, and PSNR = 10.958, which are sufficiently large distortions, the result of which is clearly visible in Figure 10.

As can be seen from the above, the existing method of steganographic hiding of text information in an audio signal based on the wavelet transform shows rather mediocre results in terms of compression resistance.

Let conduct an experimental study of the developed method and clearly see its advantage over the existing one.

Table 2 presents the results of an experimental study of the developed method for hiding text information in an audio signal based on the wavelet transform, and as will be seen below, the proposed approach significantly increases the robustness of stego-system to the deliberate and passive elimination of redundancy to distort text information.

In doing so CR = 1, we have CC = 0.9999, NRMSE = 0.0059, SNR = 36.7274, and PSNR = 63.7437, which corresponds to the high performance of the psychophysiological model of audio signal perception (masking efficiency), and CC = 1, NRMSE = 0, SNR = ∞, and PSNR = ∞ characterizes the integrity of text information extracted from the audio signal.

Very close attention should be paid to the results shown in Table 2 for CR = 20, namely CC = 0.9109, NRMSE = 0.2990, SNR = 16.3873, and PSNR = 28.3405: they characterize a strong distortion of the steganographic audio signal, but according to CC = 1, NRMSE = 0. SNR = ∞, PSNR = ∞ text information remains integrity. These results are quite remarkable, since when compressed by 20 times, the integrity of the text is preserved in full: it is this result that is significant in our study.

It should be remembered that, by analyzing the existing method, we obtained the boundary value CR = 6, and in the developed, CR = 20, with full integrity of text information in both cases. Then, we can make reasonable conclusions that by applying the developed method of steganographic hiding of text information in an audio signal, we will get a gain of 3.3 times compared to the existing method, thereby increasing the robustness of the stego-system to deliberate or passive compression of the audio signal in order to distort the embedded text information.

**Figure 11:**
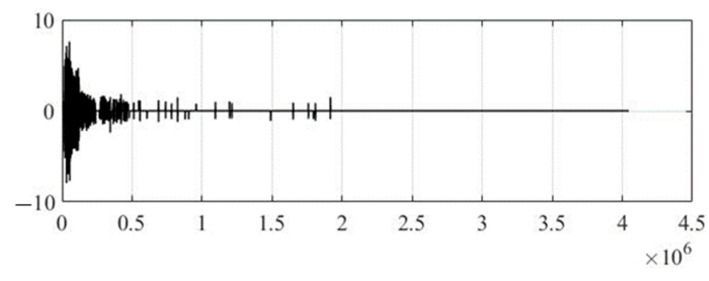
Wavelet coefficients of compressed steganocoded audio signal by 20 times with full integrity of text information (developed method).

The wavelet coefficients of the steganocoded audio signal after 20-times compression are shown in Figure 11. According to Table 2, CR = 20 is a borderline result, above which text information will be distorted.

**Figure 12:**
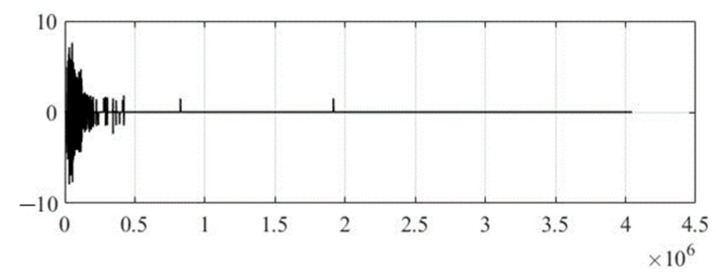
Wavelet coefficients of compressed steganocoded audio signal by 30 times (developed method).

Figure 12 shows the wavelet coefficients of the steganocoded audio signal after compression by 30 times. Given such compression, according to the values of the metrics CC = 0.9133, NRMSE = 0.3433, SNR = 21.3553, and PSNR = 34.3475, it can be concluded that text information is distorted, but comparing them with the indicators in Table 1 at the same compression level CC = 0.7453, NRMSE = 0.6893, SNR = 4.0383, and PSNR = 10.958, we will come to the conclusion that objectively, we have many times gain in the fight against distortions, all other things being equal, using the developed method of steganographic hiding of text information in an audio signal.

**Figure 13:**
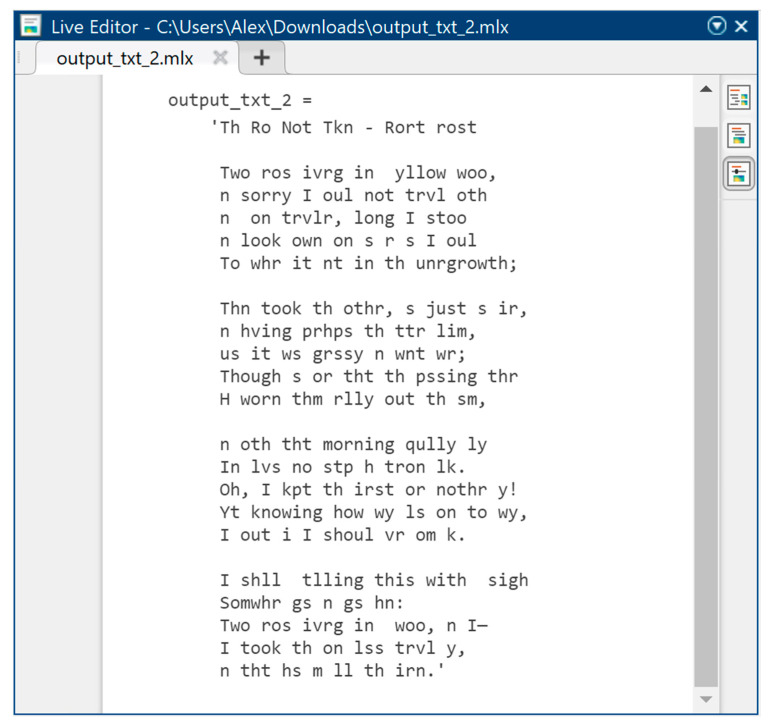
Recovered text information after 30-times compression of steganocoded audio signal (developed method).

Figure 13 shows text information with 30-fold compression of a steganocoded audio signal using the developed method. It is clearly seen that distortion occurs, but in comparison with the existing concealment method, the results of which are shown in Figure 10, we have a significant increase in the effective steganographic processing of audio signals to embed text information.

According to the results obtained in the experimental study, it is possible to draw reasonable conclusions that the proposed method of steganographic protection of text information is promising in this area and requires further research.

## 4. Conclusions

The developed method of steganographic hiding of text information in audio signal based on the wavelet transform increases the robustness of the stego-system to compression of the steganocoded audio signal, while maintaining the integrity of text information, taking into account the features of the psychophysiological model of sound perception.

The results of the obtained experimental studies confirm the hypothesis; namely, the proposal to use recursive embedding in the low-frequency region (approximating wavelet coefficients) followed by scalar product with wavelet function, which will increase the average power of hidden data. The results are given in Table 2 for CR = 20; namely, CC = 0.9109, NRMSE = 0.2990, SNR = 16.3873, and PSNR = 28.3405: they characterize a strong distortion of the steganographic audio signal, but according to CC = 1, NRMSE = 0, SNR = ∞, and PSNR = ∞, text information remains integrity. These results are quite remarkable, since when compressed by 20 times, the integrity of the text is preserved completely; this result is most significant in our study.

It should be noted that upon analyzing the existing method, which is based on embedding text information in the high-frequency component (detailed wavelet coefficients) we obtained the limit CR = 6, and in the developed, CR = 20, with full integrity of the text information in both cases. Thus, we can make reasonable conclusions that by applying the developed method of steganographic hiding of text information in audio signal, we will get a gain of 3.3 times compared to the existing method. Therefore, the resistance of the stego-system is increased by 3.3 times to deliberate or passive compression of the audio signal in order to distort the embedded text information.

The results obtained in this scientific study can be used to build systems for hiding text information in an audio file, but unlike existing methods, the developed method implements the proposed approach of the scalar product of the low-pass Daubechies filter with wavelet coefficients, where blocks of text information are already integrated. Therefore, there is an increase in the average power of low-frequency wavelet coefficients and an increase in the power of normalized ASCII codes of text information. At the same time, the developed method introduces more distortions into the signal than the existing methods, but in case of usage of audio signal with a high bitrate, we will get at the output a signal with indistinguishable quality. Because of this, we will increase by 3.3 times the resistance to intentional or unintentional compression of the output audio signal. Another disadvantage of the proposed method is that the amount of information that can be integrated into audio signal with equal measures of quality will be significantly less than in existing approaches, given the fact that the error will grow with each successive level of wavelet decomposition. Therefore, it must be emphasized that this approach will be very effective if not a large amount of data is hidden in the audio container; that is, with an increase in the amount of textual information that must be integrated into the audio signal, the effectiveness of this approach will decrease. In case of a need to hide a small amount of data, this approach will be many times more efficient than existing methods. The authors plan to consider specific quantitative assessments, at which this method will not be effective, in following scientific studies. Currently, we can conclude that when integrating text information with a volume of 740 bytes into audio signal with a volume of 7.8 MB, we get very decent results: an increase in the critical compression threshold of 3.3 times.

## Figures and Tables

**Table 1:** The efficiency indicators of the stego-system based on the wavelet transform before the implementation of the developed method.

Audio	Audio				Text			
CR	CC	NRMSE	SNR	PSNR	CC	NRMSE	SNR	PSNR
1	0.9999	0.0060	36.7794	63.0616	1	0	∞	∞
2	0.9978	0.0161	34.5934	59.8903	1	0	∞	∞
4	0.9915	0.0373	32.1520	55.4112	1	0	∞	∞
6	0.9861	0.0681	30.2330	51.6068	1	0	∞	∞
8	0.9778	0.0986	28.6479	47.9976	0.9999	0.0032	39.2855	64.1399
10	0.9676	0.1091	26.2088	43.5677	0.9792	0.1132	35.2855	58.1399
12	0.9564	0.1212	24.3915	40.6508	0.9596	0.1823	29.9880	47.8424
14	0.9407	0.1478	22.3410	37.6270	0.9378	0.2488	25.3163	40.1707
16	0.9335	0.2238	20.8485	33.2192	0.9176	0.3345	21.4123	34.2864
18	0.9215	0.2510	18.2160	31.4752	0.8958	0.3919	17.2365	30.0909
20	0.9147	0.2931	16.8701	28.2139	0.8739	0.4931	14.6098	28.4592
22	0.9032	0.3535	14.3410	26.6270	0.8524	0.4711	12.0487	24.4780
24	0.8945	0.3923	12.8485	23.2192	0.8343	0.5194	10.9584	21.0584
26	0.8874	0.4194	10.2160	19.4752	0.8130	0.5943	8.1075	17.0493
28	0.8773	0.4583	8.8701	15.2139	0.7855	0.6109	6.8347	14.7563
30	0.8632	0.4984	6.8333	11.8327	0.7453	0.6893	4.0383	10.958

**Table 2:** The efficiency indicators of the stego-system based on the wavelet transform after the implementation of the developed method.

Audio	Audio				Text			
CR	CC	NRMSE	SNR	PSNR	CC	NRMSE	SNR	PSNR
1	0.9999	0.0059	36.7274	63.7437	1	0	∞	∞
2	0.9988	0.0134	34.9352	59.5324	1	0	∞	∞
4	0.9924	0.0296	32.3301	55.1235	1	0	∞	∞
6	0.9832	0.0891	30.0373	51.0843	1	0	∞	∞
8	0.9771	0.1001	28.1047	47.4433	1	0	∞	∞
10	0.9601	0.1389	26.4402	43.5682	1	0	∞	∞
12	0.9543	0.1720	24.0921	40.8519	1	0	∞	∞
14	0.9471	0.1923	22.3241	37.8226	1	0	∞	∞
16	0.9332	0.2332	20.7392	33.5203	1	0	∞	∞
18	0.9211	0.2720	18.2974	31.2651	1	0	∞	∞
20	0.9109	0.2990	16.3873	28.3405	1	0	∞	∞
22	0.9033	0.3568	14.5520	26.3673	0.9999	0.0023	39.7464	64.9473
24	0.8912	0.3803	12.3082	23.4577	0.9734	0.1035	35.4436	58.7293
26	0.8866	0.4528	10.1325	19.3594	0.9554	0.1692	29.5677	47.2895
28	0.8723	0.4933	8.0376	15.4857	0.9307	0.2312	25.5643	40.1043
30	0.8611	0.5383	6.3243	11.5476	0.9133	0.3433	21.3553	34.3475

## Appendix A

**Listing A1:** MATLAB Code for Integrating Text Information into Wavelet Coefficients of an Audio Signal.

function [c_in,l_in,Key1,Key2,Key3] = wavtextint(x_in,T_in,wname) % Integrating text information into wavelet coefficients of an audio signal % Input parameters: % x_in - input audio signal % T_in - input text information % wname - wavelet filter % Output parameters: % c_in - wavelet coefficients with text information % l_in - levels of wavelet decomposition for reconstruction % Key1 - key for de-interleaving % Key2 - key for de-sorting % Key3 - key for extracting % Example 1 % x_in = audioread('audio_input.wav'); % T_in = fileread('text_input.txt'); % wname = 'db12'; % [c_in,l_in,Key1,Key2,Key3] = wavtextint(x_in,T_in,wname); % Example 2 % x_in = randperm(1000); % T_in = 'Text information'; % wname = 'db6'; % [c_in,l_in,Key1,Key2,Key3] = wavtextint(x_in,T_in,wname); % % O. Lavrynenko 08-December-2021. % Characters in ASCII codes ASCII = double(unicode2native(T_in)); % Interleaving k = randperm(length(ASCII)); ASCII_rand = ASCII(k); % Key 1 for de-interleaving [~,Key1] = sort(k); % Sorting [ASCII_sort,k] = sort(ASCII_rand); % Key 2 for de-sorting [~,Key2] = sort(k); % Normalization ASCII_norm = ASCII_sort/127; % Wavelet filters for decomposition [Lo_D,Hi_D] = wfilters(wname,'d'); % Maximum level of wavelet decomposition lev = fix(log2(length(x_in)/(length(Lo_D)-1))); % Maximum level of integration lev_int = fix(log2(length(x_in)/length(ASCII_norm))); % Division into blocks lb = ceil(length(ASCII_norm)/(lev_int)); ASCII_zer = [ASCII_norm,zeros(1,lb*(lev_int)-length(ASCII_norm))]; ASCII_blocks = reshape(ASCII_zer,[lb,lev_int]); % Initialization s = size(x_in); x_in = x_in(:).'; Key3 = []; c_in = []; l_in = zeros(1,lev+2,'like',real(x_in([]))); l_in(end) = length(x_in); % Wavelet decomposition for k = 1:lev % Single-level 1-D discrete wavelet transform [x_in,d_in] = dwt(x_in,Lo_D,Hi_D); % Integration if k<=lev_int [d_sort,i] = sort(abs(d_in)); i = find(d_sort>mean(d_sort)/2 & d_sort<mean(d_sort)*2); i = i(1:lb); d_in(i) = ASCII_blocks(1:lb,k); % Key 3 for extraction Key3 = [Key3 i']; end c_in = [d_in c_in]; l_in(lev+2-k) = length(d_in); end % Wavelet coefficients of the last level of decomposition c_in = [x_in c_in]; l_in(1) = length(x_in); % Transpose if s(1)>1 c_in = c_in.'; l_in = l_in.'; end

**Listing A2:** MATLAB Code for Reconstructing an Audio Signal from Wavelet Coefficients with Text Information.

function x_out = wavaudiorec(c_in,l_in,wname) % Reconstructing an audio signal from wavelet coefficients with text information % Input parameters: % c_in - wavelet coefficients with text information % l_in - levels of wavelet decomposition for reconstruction % wname - wavelet filter % Output parameters: % x_out - audio signal with text information % Example 1 % x_in = audioread('audio_input.wav'); % T_in = fileread('text_input.txt'); % wname = 'db12'; % [c_in,l_in,Key1,Key2,Key3] = wavtextint(x_in,T_in,wname); % x_out = wavaudiorec(c_in,l_in,wname); % Example 2 % x_in = randperm(1000); % T_in = 'Text information'; % wname = 'db6'; % [c_in,l_in,Key1,Key2,Key3] = wavtextint(x_in,T_in,wname); % x_out = wavaudiorec(c_in,l_in,wname); % % O. Lavrynenko 08-December-2021. % Determine whether input is column vector IsColumn = iscolumn(c_in); % Transpose if IsColumn c_in = c_in.'; l_in = l_in.'; end % Wavelet filters for reconstruction [Lo_R,Hi_R] = wfilters(wname,'r'); % Initialization x_out = c_in(1:l_in(1)); % Wavelet reconstruction for p = length(l_in)-2:-1:1 % Detail coefficients d_out = detcoef(c_in,l_in,p); % Single-level inverse discrete 1-D wavelet transform x_out = idwt(x_out,d_out,Lo_R,Hi_R,l_in((length(l_in)+1)-p)); end % Transpose if IsColumn x_out = x_out.'; end

**Listing A3:** MATLAB Code for Extracting Text Information from Wavelet Coefficients of an Audio Signal.

function [T_out,c_out,l_out] = wavtextext(x_out,wname,Key1,Key2,Key3) % Extracting text information from wavelet coefficients of an audio signal % Input parameters: % x_out - audio signal with text information % wname - wavelet filter % Key1 - key for de-interleaving % Key2 - key for de-sorting % Key3 - key for extracting % Output parameters: % T_out - output text information % c_out - wavelet coefficients with text information % l_out - levels of wavelet decomposition for reconstruction % Example 1 % x_in = audioread('audio_input.wav'); % T_in = fileread('text_input.txt'); % wname = 'db12'; % [c_in,l_in,Key1,Key2,Key3] = wavtextint(x_in,T_in,wname); % x_out = wavaudiorec(c_in,l_in,wname); % [T_out,c_out,l_out] = wavtextext(x_out,wname,Key1,Key2,Key3); % Example 2 % x_in = randperm(1000); % T_in = 'Text information'; % wname = 'db6'; % [c_in,l_in,Key1,Key2,Key3] = wavtextint(x_in,T_in,wname); % x_out = wavaudiorec(c_in,l_in,wname); % [T_out,c_out,l_out] = wavtextext(x_out,wname,Key1,Key2,Key3); % % O. Lavrynenko 08-December-2021. % Wavelet filters for decomposition [Lo_D,Hi_D] = wfilters(wname,'d'); % Maximum level of wavelet decomposition lev = fix(log2(length(x_out)/(length(Lo_D)-1))); % Initialization s = size(x_out); x_out = x_out(:).'; [numRows,numCols] = size(Key3); ASCII_ext = []; c_out = []; l_out = zeros(1,lev+2,'like',real(x_out([]))); l_out(end) = length(x_out); % Wavelet decomposition for k = 1:lev % Single-level 1-D discrete wavelet transform [x_out,d_out] = dwt(x_out,Lo_D,Hi_D); % Extraction if k<=numCols i = d_out(Key3(1:numRows,k)); % ASCII codes ASCII_ext = [ASCII_ext i']; end c_out = [d_out c_out]; l_out(lev+2-k) = length(d_out); end % Wavelet coefficients of the last level of decomposition c_out = [x_out c_out]; l_out(1) = length(x_out); % Transpose if s(1)>1 c_out = c_out.'; l_out = l_out.'; end % Combining blocks ASCII_deblocks = reshape(ASCII_ext,[1,numRows*numCols]); ASCII_deblocks(ASCII_deblocks==0) = []; % De-normalization ASCII_denorm = round(ASCII_deblocks*127); % De-sorting ASCII_desort = ASCII_denorm(Key2); % De-interleaving ASCII_derand = ASCII_desort(Key1); % ASCII codes to characters T_out = native2unicode(ASCII_derand);
